# 

**Published:** 1852-04

**Authors:** 


### CONTENTS

OF THE

### MEDICAL EXAMINER.

NEW SERIES—NO. LXXXVIII.—APRIL, 1852.

### ORIGINAL COMMUNICATIONS.

Medical Topography, with Observations on the Epidemic Dysentery of Lancaster County. With a Cut. By D. H. Agnew, M. D., Philadelphia, - - - - - - - 205

Laryngotomy successfully performed in a case of Foreign Body in the Larynx. By G. R. Morehouse, M. D., Philadelphia, - 215

Surgical Cases communicated to D. Gilbert, M. D., Professor of Surgery in the Medical Department of Pennsylvania College, Philadelphia, - - - - - - - 218

An account of the operation of Tracheotomy for the relief of Croup—of Lithotomy under etherisation, and of Amputation of the Left Half of the Upper Jaw. By Henry H. Smith, M. D., Surgeon to the St. Joseph's Hospital, Philadelphia, - - 222

A Case of Poisoning from the external application of the Cocculus Indicus. Reported by William B. Thompson, Sr. House Surgeon, Emigrant's Hospital, Ward's Island, - - 227

Remarks on Epithelial Tumors and Cancer of the Skin. By J. Da Costa, M. D., of Philadelphia, - - - - 229
Philadelphia County Medical Society. Extra Meeting, March 9th, 1852. Discussion on Anæsthesia, - - - - 243

### BIBLIOGRAPHICAL NOTICES.

Illustrated Manual of Operative Surgery and Surgical Anatomy. By MM. Cl. Bernard and Ch Huette; edited with notes and additions, and adapted to the use of the American Medical Student, by W. H. Van Buren, M. D., Surgeon to Bellevue Hospital, &c., and C. E. Isaacs, M. D., Demonstrator of Anatomy College Physicians and Surgeons, New York. Illustrated with steel engravings, from drawings after nature, by M. J. Léveillé, designed to serve as a companion to the ordinary text books of Surgery, - - - - - - 260
Outlines of the Nerves, with short descriptions, designed for the use of Medical Students; and Outlines of the Arteries, with short descriptions, designed for the use of Medical Students. By John Neill, A. M., M. D., Surgeon to Will's Hospital, Demonstrator of Anatomy in the University of Pennsylvania, - 263

### MEDICAL NEWS.

Philadelphia County Medical Society. Election of Officers and Delegates, - - - - - - - 264
Delaware County Medical Society. Election of Officers, - - 265
Delegates to the American Medical Association, appointed by the College of Physicians of Philadelphia, - - - 265
Physicians' Prescriptions, - - - - - - - 265
Appointment in the University of New York, - - - 265
Itch Insect, - - - - - - - - - 266
Spread of Disease and Fever in Ireland, - - - - - 266
American Medical Association, - - - - - - 266

Discontinuance of the British American Medical and Physical Journal, - - - - - - - 267
Etherization in Parturition Nineteen Centuries ago, - - - 267
Medical Circulars, - - - - - - - 267
Remarkable Case of Inanition, - - - - - 268
University of Louisville Lawsuit, - - - - - 268
Resignation of Dr. John Bell, - - - - - 268
Relative Mortality of the States, - - - - - 268
New Lights and New Livers, - - - - - 269

### RECORD OF MEDICAL SCIENCE.

### SURGERY.

Wound of the Stomach with Protrusion, - - - - 271

